# Pulmonary and chest wall function in obese adults

**DOI:** 10.1038/s41598-023-44222-3

**Published:** 2023-10-18

**Authors:** Antonella Lo Mauro, Gabriella Tringali, Franco Codecasa, Laura Abbruzzese, Alessandro Sartorio, Andrea Aliverti

**Affiliations:** 1 Dipartimento di Elettronica, Informazione e Bioingegneria, Politecnico di Milano, Piazza L. Da Vinci, 20133 Milan, Italy; 2 Experimental Laboratory for Auxo-Endocrinological Research, Istituto Auxologico Italiano, IRCCS, Piancavallo-Verbania, Italy; 3 Division of Pneumological Rehabilitation, Istituto Auxologico Italiano, IRCCS, Piancavallo-Verbania, Italy; 4 Division of Eating and Nutrition Disorders, Istituto Auxologico Italiano, IRCCS, Piancavallo-Verbania, Italy; 5 Experimental Laboratory for Auxo-Endocrinological Research, Istituto Auxologico Italiano, IRCCS, Milan, Italy

**Keywords:** Diseases, Medical research

## Abstract

Obesity is frequently associated with breathing disorders. To investigate if and how the highest levels of obesity impact respiratory function, 17 subjects with obesity (median age: 49 years; BMI: 39.7 kg/m^2^, 8 females) and 10 normal-weighted subjects (49 years; 23.9 kg/m^2^, 5 females) were studied. The abdominal volume occupied 41% in the obese group, being higher (*p* < 0.001) than the normal-weighted group (31%), indicating accumulation of abdominal fat. Restrictive lung defect was present in 17% of subjects with obesity. At rest in the supine position, subjects with obesity breathed with higher minute ventilation (11.9 L/min) and lower ribcage contribution (5.7%) than normal weighted subjects (7.5 L/min, *p* = 0.001 and 31.1%, *p* = 0.003, respectively), thus indicating thoracic restriction. Otherwise healthy obesity might not be characterized by a systematic restrictive lung pattern. Despite this, another sign of restriction could be poor thoracic expansion at rest in the supine position, resulting in increased ventilation. Class 3 obesity made respiratory rate further increased. Opto-electronic plethysmography and its thoraco-abdominal analysis of awake breathing add viable and interesting information in subjects with obesity that were complementary to pulmonary function tests. In addition, OEP is able to localize the restrictive effect of obesity.

## Introduction

Obesity is one of the major health concerns, as it has reached a worldwide epidemic dimension. According to the World Health Organization, in 2016 ~ 1.9 billion adults worldwide (~ 39%) were overweight, with 650 million (13%) obese. Such worldwide prevalence of obesity results in an increase in the prevalence, morbidity, and clinical presentation, among others, of many respiratory diseases. With over 4 million people dying each year as a result of being overweight or obese in 2017, the global burden of obesity has grown to epidemic proportions^1^.

According to body mass index (BMI), obesity is usually subdivided into 3 categories: class 1: BMI of 30 to < 35; class 2: BMI of 35 to < 40 and class 3: BMI ≥ 40^2,3^. The latter is sometimes categorized as “severe” obesity and it can contribute to the development of several serious concomitant diseases. These comprise metabolic syndrome, type 2 diabetes, heart disease, hypertension, atherosclerosis, certain cancers, osteoarthritis, depression, and breathing issues. The respiratory symptoms and complications, obesity is frequently associated with, include breathing at lower lung volumes, decreased thoracic and lung compliance, increased respiratory resistance secondary from the reduction in lung volumes, reduction in respiratory muscle strength, heterogeneity of ventilation distribution, increased pulmonary diffusion and hypercapnic respiratory failure^4,5^. In addition to such augmented elastic load due to the mass burden on the chest wall, subjects with obesity have also to overcome the higher resistive load. Supine positioning even exacerbates these features because of the positional adipose tissue redistribution, with abdominal content shifting cranially, and mediastinal weight^6^ that reduces the functional residual capacity (FRC) while passively stretching the diaphragm^7,8^. As a result, respiratory muscles have to cope with increased work of breathing therefore impacting on thoraco-abdominal kinematics.

Opto-electronic plethysmography (OEP) provides a very effective and accurate evaluation of thoraco-abdominal kinematics in different postures and conditions^9–14^. OEP was already used to evaluate the thoraco-abdominal kinematics of children^15^, adolescents^16^ and adults with obesity up to BMI ~ 50 kg/m^2^^17–21^. However, the marker configurations and the corresponding geometrical models of OEP were designed and validated on normal-weighted subjects^22,23^. The density of the mesh of OEP markers might be not thick enough for the increased chest wall dimension of subjects with obesity, with the risk of lower accuracy of OEP. At this time, OEP was validated only in women with obesity in seated position and during quiet breathing^21^. In addition to dynamic volume variation, OEP also provides accurate static thoraco-abdominal volumes and it was proved to highlight altered chest wall geometry and/or dimension in relation to possible modified breathing thoraco-abdominal pattern^24–27^.

In a small set of obese and lean adult subjects, we aimed to evaluate if and how the highest levels of obesity impact the pulmonary and chest wall function, by using standard pulmonary function tests (PFT) and OEP technique. Our main hypothesis was that accurate measurement of thoraco-abdominal volume might provide additional and/or complementary information regarding any possible restrictive effect of obesity. The second aim was to evaluate the effects of posture (i.e. supine vs. seated) on spirometric parameters, lung capacities, and ventilatory parameters at rest. Possible correlations between the abdominal dimension and the supine fall of FRC were investigated. Thirdly, we aimed to compare different obesity levels, namely class 2 and class 3, in terms of lung and thoraco-abdominal volumes as well as ventilatory pattern and chest wall dimensions. Finally, we aimed to confirm the validity of the OEP technique in seated and supine positions not only during quiet breathing but also during maximal maneuvers in a population including both females and males.

## Materials and methods

### Subjects and study protocol

Adults with obesity attending a 3-week in-patient multidisciplinary body weight reduction program were enrolled in the study. Lacking predicted values or equations for the opto-electronic plethysmography data (see below), a group of healthy normal weighted subjects was recruited among colleagues and friends of the authors and acquired only with opto-electronic plethysmography to create the control group for these data. The normal weighted subjects were chosen to reflect the distribution of the groups with obesity in terms of gender and age. Stable condition and absence of severe cardio-respiratory pathologies were the inclusion criteria for both groups. Informed consent was obtained from all study participants. The procedures of the investigation were approved by the ethics committee of Istituto Auxologico Italiano, Milan, Italy (research code: 01C307-2013; acronym: POSTVOLOB) and were performed in agreement with the recommendations outlined in the Helsinki Declaration.

### Anthropometry, body composition, and chest wall geometry

Standard measures of height, weight, and body mass index (calculated as body weight/height squared) were made together with the assessment of fat mass, fat-free mass, and thoraco-abdominal perimeters, areas, and volumes.

Body composition was measured using a multifrequency tetrapolar impedance meter (BIA, Human-IM Scan, DS-Medigroup, Milan, Italy) with a delivered current of 800 μA at a frequency of 50 kHz. To reduce errors of measurement, special care was paid to the standardization of the variables known to affect measurement validity, reproducibility, and precision. Measurements were performed according to the method of Lukaski^28^ (i.e.: after 20 min of resting in a supine position with arms and legs relaxed and not in contact with other body parts) and in strictly controlled conditions.

Waist circumference was measured midway between the lower rib margin and the superior anterior iliac spine using a horizontally applied non-stretch tape. Hip circumference was measured around the widest portion of the buttocks, with the same non-stretch tape parallel to the floor.

Thoraco-abdominal volumes were measured by opto-electronic plethysmography (OEP, Smart System BTS, Milan, Italy), both in the seated and supine positions. Eight video cameras tracked the movement of retro-reflective markers (89 in the seated position, 52 in the supine position^9^) placed anteriorly and posteriorly (only in the seated position) over the chest wall from the clavicles to the pubis. The total chest wall volume (V_CW_) was calculated by applying Gauss’s theorem to the three-dimensional coordinates of the markers. The chest wall was modeled as being composed of two compartments: the ribcage (V_RC_, volume enclosed by the clavicles, and the lower costal margin of the rib cage where the diaphragm is apposed) and the abdomen (V_AB_, volume enclosed by the lower costal margin of the rib cage and the iliac crests). Ribcage and abdominal volumes were considered as absolute values but also expressed as a percentage of total chest wall volume.

### Spirometry, lung capacities, and maximal respiratory pressures

Forced vital capacity (FVC), forced expiratory volume in one second (FEV_1_), their ratio (FEV_1_/FVC) as well as forced expiratory flow at 25 and 75% of the pulmonary volume (FEF_25–75_) were determined with the subject in standing position (Med-Graphics CPX/D, Medical Graphic Corp., USA). Lung capacities (*i.e.*: functional residual capacity (FRC), total lung capacity (TLC) and residual volume (RV), inspiratory capacity (IC = TLC-FRC), vital capacity (VC = TLC-RV) and expiratory reserve volume (ERV = FRC-RV)) were measured by the nitrogen washout technique (Vmax series 22, SensorMedics, Yorba Linda, CA, USA) in seated and supine position. Maximal inspiratory (MIP) and expiratory (MEP) pressures were measured at the mouth in both postures by a piezo-resistive pressure transducer (RCEM250DB, Sensortechnics, Puchheim, Germany). All tests were carried out by the same technicians according to the European Respiratory Society (ERS) guidelines^29–31^. Reference equations for spirometry and lung volumes were derived from the Global Lung Function Initiative calculators for spirometry^32^ and lung volume^33^ and also from the Lung volumes and forced ventilatory flows.

Lung restriction was defined according to the ERS criteria^34^. A significant restriction was identified when the percentage value of TLC was below the 5th percentile (corresponding to 1.64 relative standard deviation) and FEV_1_/VC ratio was normal.

The predictive equations reviewed by Evans and Whitelaw in 2009 were considered for respiratory muscle strength because they also proposed a lower limit of normal (LLN) values^35^.

### Ventilatory and thoraco-abdominal pattern

Being a motion analysis system, OEP provides the 3D coordinate of the markers over time, therefore providing chest wall kinematics during breathing*.* Once instrumented and after a period of familiarization, subjects were asked to breathe normally for at least 5 min and to perform two vital capacities maneuvers at the end. This test was started in a seated position and then repeated in the supine position. Starting from chest wall volume traces, an average breath was obtained by at least one minute of spontaneous awake quiet breathing. From this averaged breath, tidal volume, breathing frequency, and minute ventilation (as the product of tidal volume and breathing frequency) were determined. Ribcage and abdominal tidal volumes were also calculated and expressed as percentage contributions to tidal volume.

### OEP validation

To evaluate the agreement between the chest wall and lung volume variations, the flow at the mouth was measured (and then integrated) simultaneously with OEP using a pneumotacograph (Hans-Rudolph, Kansas, USA). Subjects were therefore equipped with a mouthpiece and a noseclip. The relationships between the two clinical measurements of volumes were estimated using a linear regression analysis as well as a Bland and Altman plot, on a breath-by-breath analysis, considering the integrated volume as the gold standard. The percentage error was also calculated as the difference between chest wall volume and the integrated flow, divided by the integrated flow, multiplied by 100%. The validation was completed in both postures and during quiet breathing, inspiratory capacity, and expiratory vital capacity of the best maneuver.

### Statistical analysis

The effect of obesity classes on anthropometry, spirometry, lung capacities, maximal pressures, chest wall volumes, and ventilatory pattern was tested using One Way Analysis of Variance (when the normality test was passed) or the Wilcoxon–Mann–Whitney U test (when the normality test failed) with obesity class as independent factor. The Wilcoxon Signed Rank Test was used to test the postural effect on spirometry, lung capacities, and maximal pressures, with posture as an independent factor. The same test was used to compare the percentage values of spirometric and absolute lung volumes, with the prediction equation as an independent factor.

When the control group was included in the analysis, the Kruskal–Wallis One Way Analysis of Variance on Ranks was used in case the normal distribution was not met.

Post-hoc tests were based on Holm–Sidak’s or Dunn's method for parametric and non-parametric analysis, respectively.

To verify if the abdominal dimension impacted the supine FRC fall, a correlation analysis was performed between FRC (considering both the absolute value in the supine position, but also the percentage of the seated FRC) and ribcage volume (considering its absolute value in the supine position, its percentage contribution to chest wall in the supine position and its ratio with the seated values).

Data in the text are presented as median (25th percentile–75th percentile). The level of significance was set at *p* < 0.05.

### Sample size calculation

Because we hypothesized thoraco-abdominal volume to add complementary information on the restrictive effect of obesity, we used the percentage contribution of the ribcage to tidal volume assessed by OEP (%RC) published in Table 3 by de Melo Barcelar et al.^21^. The appropriate sample size for detecting a difference between the means of two independent samples, namely obese (mean %RC = 38.7% with a standard deviation of 9.8) and lean women (mean %RC = 64.4% with a standard deviation of 12.7), was then calculated. The difference between these two independent means provided an effect size of 2.265 that, with a type-1 error probability α = 0.05, a power (1-β, with β being type-2 error probability) = 0.95, and an allocation ratio 1:1, resulted in a sample size of 12 subjects (6 obese and 6 lean subjects). The power analysis was performed in G*Power 3.1.9.4 software.

## Results

### Subjects, anthropometry, body composition and chest wall geometry

Seventeen adult subjects with obesity accepted to participate: 10 were obese class 2 (*i.e.*: BMI ranging between 35 and 40, 5 females) and 7 were obese class 3 (*i.e.*: BMI ≥ 40, 3 females), who differed obviously in terms of BMI (Table 1). Ten healthy normal weighted subjects (4 females) were gathered as the control group, being similar in age (49 (33.7–55.7) years, *p* = 0.313) and height (173 (170.5–175.7) cm, *p* > 0.05), but lighter (73 (70.2–85.0) kg, *p* < 0.001) than subjects with obesity, resulting into a lower BMI (24 (22.6–27.7) kg/cm^2^, *p* < 0.001). Fat mass and fat-free mass were similar between the two groups of subjects with obesity (Table 1).

**Table 1:** Anthropometric data of obese patients.

Parameter	Unit	Overall			Class 2			Class 3			*p*-value
		Median	25th p	75th p	Median	25th p	75th p	Median	25th p	75th p	
Age	Years	49.0	35.0	51.0	51.0	47.8	52.5	**41.0**	32.5	46.5	0.123
Height	cm	165.0	160.0	168.0	167.5	162.8	174.0	**160.0**	152.5	166.5	0.121
Weight	kg	112.0	98.0	123.0	108.0	99.4	120.3	**113.0**	103.7	129.5	0.387
BMI	kg/cm^2^	39.7	38.7	41.5	38.7	37.3	39.0	**45.7**	41.4	50.2	< 0.001
FM	kg	50.6	47.8	57.9	50.5	42.2	54.9	**52.4**	49.1	62.1	0.092
FM	%	43.3	39.0	50.0	41.2	35.4	49.3	**47.5**	41.5	50.2	0.271
FFM	kg	69.3	53.2	82.6	62.9	53.4	81.8	**69.8**	51.9	84.6	0.810
FFM	%	52.9	48.8	60.9	58.8	50.7	64.7	**50.8**	47.9	54.8	0.090

*BMI* body mass index, *FFM* fat free mass, *FM* fat mass, *25p* 25th percentile, *75p* 75th percentile.

Waist circumference was similar between class 2 (125.5 (121.3–132.5) cm) and class 3 (140.0 (123.0–148.5) cm; *p* = 0.172), while hip circumference was higher in the latter (131.0 (129.0–145.5) cm; *p* = 0.019) compared to the former (125.0 (123.0–130.5) cm), resulting in a similar waist/hip ratio (class2: 1.02 (0.96–1.07); class3: 0.98 (0.91–1.05); *p* = 0.494), while the waist-stature ratio was higher in class3 (0.86 (0.79–0.92); class2: (0.77 (0.72–0.78), *p* = 0.04).

Taking into account all the above results, subjects were characterized by abdominal obesity, as they all met the specific criteria (i.e.: absolute waist circumference > 102 in men and > 88 cm in women^36^; waist–hip ratio > 0.9 for men and > 0.85 for women^37^ and waist-stature ratio > 0.5 for adults under 40 and > 0.6 for adults over 50).

In the seated position, total chest wall volume was significantly lower in the control group (24.1 (21.8–32.1) L, *p* < 0.001) compared to both class 2 (40.4 (37.0–42.7) L) and class 3 (51.4 (34.6–56.2) L), but not within the two obesity classes. The abdominal volume occupied 31%, 40%, and 42% of the chest wall volume respectively in normal weighted, class 2 and class 3 subjects, being significantly lower in the control group (*p* < 0.001) with no difference between the two subgroups of subjects with obesity (*p* = 0.816). In the supine position, the abdominal volume contribution increased at 43%, 52%, and 54% in normal weighted, class 2 and class 3 subjects, respectively, being significantly lower in the control group (*p* < 0.001) with no difference between the two subgroups of subjects with obesity (*p* = 0.897).

### OEP validation

**Figure 1:**
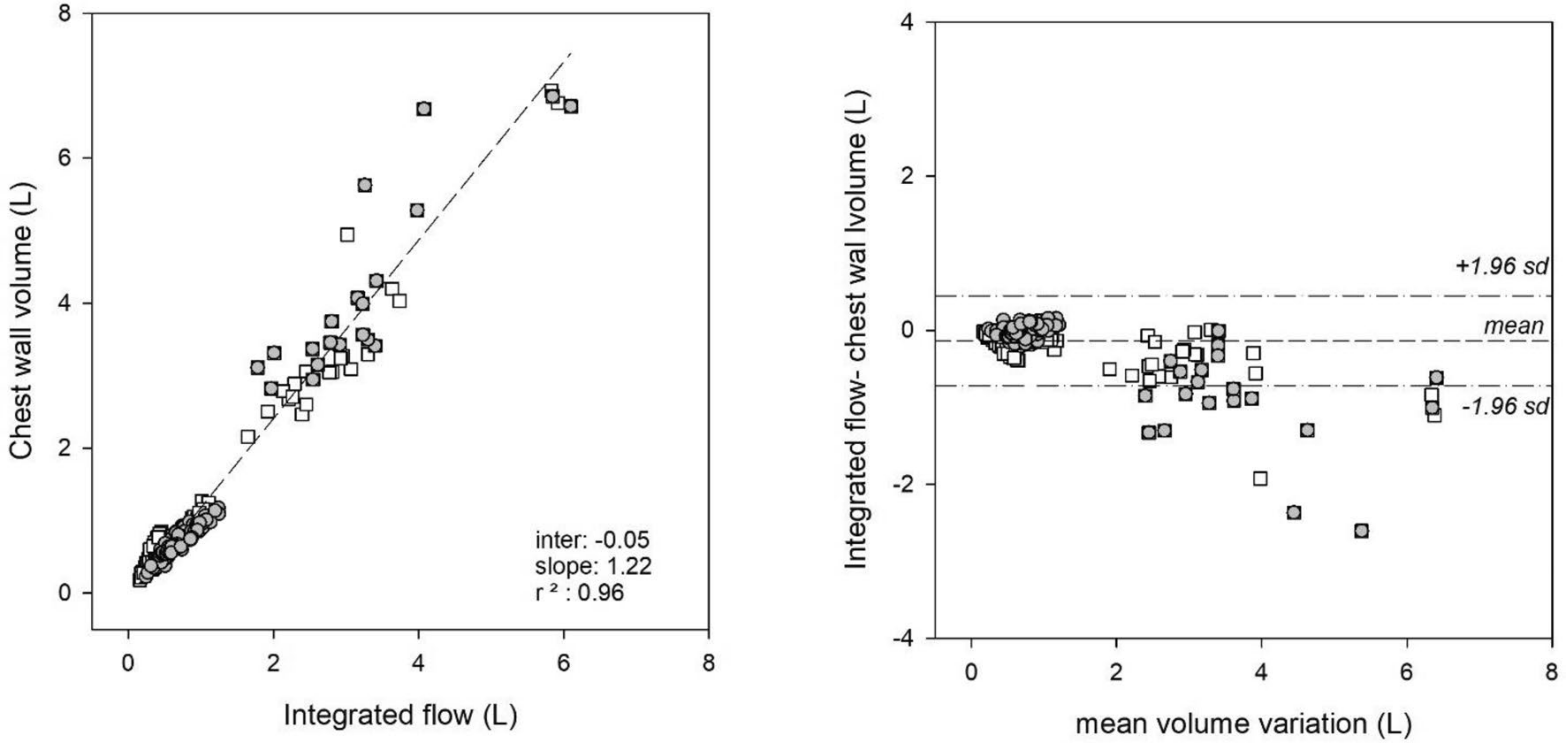
Relationship between volume variation during quiet spontaneous breathing and maximal maneuvers measured by integration of airflow measured at the mouth by a pneumotachograph (*x-axis*) and by opto-electronic plethysmography (*y-axis*) in seated (grey circles) and supine (white square) position. The short-dashed line represents the linear regression among all the considered breaths (left panel). Bland–Altman plot of the same breaths (grey circles: seated position; white square: supine position).

Pooling together all the selected breaths (both at rest and during maximal maneuvers; both in seated and supine position), a very good correlation (the coefficient of determination = 0.96) was found between the two systems, with the OEP system overestimating the integrated flow (slope of the interpolation line = 1.22), as shown in Fig. 1. The Bland & Altman plot showed that at rest the two methods provided consistent results (Fig. 1). Indeed, at rest during quiet breathing the OEP system overestimated the integrated flow by 52 (13–96) mL, corresponding to a percentage error of 8.5 (2.4–17.2) %. During maximal maneuvers, the overestimation rose to 0.59 (0.31–0.89) L, corresponding to a percentage error of 20.2 (10.2–30.7) %. Posture (*p* = 0.139) and BMI (p = 0.159) did not affect the median percentage error.

### Spirometry, lung capacities, and maximal respiratory pressures

Spirometry, lung capacities, and maximal respiratory pressures were not influenced by the obesity degrees both in seated (Table 2) and supine (Table 3) positions. FEV_1_/FVC was not lower than the 5th percentile, therefore excluding the presence of obstructive alteration^34^. Lung restriction was also excluded, as TLC was within normal values. Overall, ERV was decreased (both considering the percentage value and the z-score). RV was higher while FRC was relatively well preserved. Vital capacity was reduced (i.e.: z-score <  − 1.64) in only one patient. The same patient showed reduced FRC, TLC, and RV. A supine fall occurred for FRC, while TLC and RV remained stable, with consequent reduction of ERV and increased IC (Table 3).

**Table 2:** Spirometry, lung capacities and maximal pressures of obese patients in seated position.

Parameter	Unit	Overall			Class 2			Class 3			*p*-value
		Median	25th p	75th p	Median	25th p	75th p	Median	25th p	75th p	
FEV_1_	liters	2.83	2.66	3.48	2.83	2.66	4.20	2.83	2.69	2.92	0.587
	% pred	95.8	90.5	104.8	94.7	88.6	107.2	96.6	95.7	97.6	0.733
	z-score	− 0.34	− 0.74	0.37	− 0.41	− 0.93	0.55	− 0.26	− 0.35	− 0.19	0.717
FVC	liters	3.51	3.18	4.63	3.49	3.12	5.11	3.51	3.25	4.16	0.807
	% pred	96.6	86.5	98.9	94.8	86.2	104.8	97.5	91.1	98.3	0.970
	z-score	− 0.26	− 0.98	− 0.09	− 0.42	− 1.03	0.34	− 0.19	− 0.70	− 0.13	0.939
FEV1/FVC	%	83.50	81.33	85.50	82.75	81.85	84.83	83.70	80.38	85.98	0.801
	% pred	102.6	100.7	105.5	103.8	101.2	106.1	101.3	98.6	103.7	0.465
	z-score	0.34	0.10	0.76	0.51	0.16	0.85	0.18	− 0.21	0.53	0.445
FEF_25-75_	liters/sec	3.50	2.94	4.54	3.92	3.13	4.69	3.14	2.83	3.68	0.274
	% pred	114.7	103.7	132.0	124.6	101.3	146.6	109.3	105.9	116.4	0.264
	z-score	0.50	0.13	0.95	0.76	0.03	1.33	0.32	0.21	0.56	0.387
FRC	liters	2.59	2.22	3.24	3.10	2.28	3.33	2.55	2.29	2.74	0.220
	% pred	102.2	78.7	116.5	97.2	80.1	117.9	102.2	86.3	113.6	0.987
	z-score	0.10	− 1.07	0.78	− 0.13	− 1.01	0.84	0.10	− 0.68	0.64	0.895
TLC	liters	5.41	4.68	6.42	5.98	4.50	6.91	4.88	4.69	5.92	0.269
	% pred	95.8	89.2	110.3	98.0	85.3	111.2	95.8	92.4	104.6	0.933
	z-score	− 0.35	− 0.92	0.86	− 0.18	− 1.31	0.93	− 0.35	− 0.65	0.38	0.947
RV	liters	1.75	1.46	2.22	1.92	1.50	2.46	1.75	1.48	1.78	0.282
	% pred	127.0	101.4	143.8	120.1	97.0	146.2	127.0	117.6	136.0	0.720
	z-score	0.86	0.04	1.27	0.67	− 0.11	1.54	0.86	0.55	1.11	0.749
RV/TLC	liters	31.33	27.57	34.93	32.14	28.22	33.85	30.74	27.65	36.08	0.857
	% pred	123.7	114.8	134.3	116.1	113.3	125.0	132.5	126.2	135.2	0.423
	z-score	1.01	0.67	1.38	0.72	0.52	1.10	1.15	0.95	1.38	0.561
ERV	liters	0.91	0.78	1.09	0.93	0.79	1.20	0.81	0.77	1.06	0.365
	% pred	83.0	77.6	91.8	85.3	78.7	91.4	79.2	71.9	90.5	0.636
	z-score	− 0.41	− 0.66	− 0.19	− 0.39	− 0.54	− 0.20	− 0.59	− 0.80	− 0.22	0.558
IC	liters	2.64	2.15	3.54	2.77	2.20	3.64	2.38	2.22	3.09	0.499
	% pred	97.6	81.7	109.6	97.5	77.1	112.7	97.6	89.1	103.0	0.874
	z-score	− 0.13	− 1.02	0.54	− 0.16	− 1.28	0.74	− 0.13	− 0.60	0.18	0.882
VC	liters	3.38	3.01	4.64	3.67	3.09	5.07	3.38	3.07	4.16	0.363
	% pred	84.5	78.4	96.5	86.0	77.6	100.8	83.5	81.8	93.4	0.784
	z-score	− 1.27	− 1.71	− 0.29	− 1.15	− 1.76	0.06	− 1.33	− 1.49	− 0.54	0.784
MIP	cmH_2_O	− 64.3	− 83. 8 − 43.9	− 483.89	− 58.37	− 82.5440.24	− 40.1482.54	− 69.04	− 101.8049.82	− 49.82101.80	0.258
	% pred	69.3	50.2	83.4	66.6	48.2	82.5	72.4	56.3	97.1	0.390
	% pred LLN	130.3	104.0	154.1	131.0	102.4	152.5	130.3	108.9	180.3	0.374
MEP	cmH_2_O	94.37	64.64	107.72	96.08	71.25	103.85	85.21	57.04	114.60	0.642
	% pred	73.0	60.9	93.4	77.7	68.9	94.0	60.9	54.9	83.0	0.201
	% pred LLN	117.3	83.3	138.9	124.0	106.4	137.8	102.7	74.0	135.7	0.380

*FEV*_*1*_ forced expiratory volume in the 1 s, *FVC* forced vital capacity, *FEF*_*25-75*_ forced expiratory flow at 25 and 75% of the pulmonary volume, *FRC* functional residual capacity, *LLN* lower limit of normal, *TLC* total lung capacity, *RV* residual volume, *ERV* expiratory residual volume, *IC* inspiratory capacity, *VC* vital capacity, *MIP* maximal inspiratory pressure, *MEP* maximal expiratory pressure, *%pred* percentage of predicted value, *25p* 25th percentile, *75p* 75th percentile.

**Table 3:** Lung capacities and maximal pressures of obese patients in supine position.

Parameter	Unit	Overall			Class 2			Class 3			2 versus 3	Versus seated
		Median	25th p	75th p	Median	25th p	75th p	Median	25th p	75th p	*p*-value	*p*-value
FRC	liters	2.29	2.02	2.61	2.50	2.22	2.71	**2.04**	1.90	2.23	0.144	0.003
	% seated	82.2	90.8	79.0	84.7	89.6	80.5	**80.6**	98.4	73.8	0.910	–
TLC	liters	5.14	4.71	6.18	5.89	4.76	6.93	**5.00**	4.68	5.21	0.145	0.398
	% seated	97.3	102.4	94.5	98.5	102.6	94.6	**96.2**	101.1	94.0	0.898	–
RV	liters	1.67	1.55	1.90	1.78	1.59	2.01	**1.61**	1.36	1.68	0.111	0.489
	% seated	92.8	107.2	87.3	95.3	109.0	91.2	**88.7**	92.2	85.1	0.386	–
ERV	liters	0.58	0.45	0.73	0.58	0.48	0.77	**0.54**	0.42	0.60	0.676	< 0.001
	% seated	58.8	68.7	54.7	61.3	69.3	54.7	**58.5**	63.9	55.8	0.550	–
IC	liters	3.05	2.55	3.41	3.14	2.56	4.19	**2.97**	2.65	3.10	0.301	0.002
	% seated	111.9	122.8	107.7	110.1	120.9	108.7	**115.9**	122.6	107.6	0.995	–
VC	liters	3.47	3.13	4.02	3.61	3.18	5.11	**3.38**	3.06	3.70	0.294	0.871
	% seated	97.9	102.4	95.5	97.7	102.3	94.4	**98.6**	101.6	97.0	0.681	–
MIP	cmH_2_O	− 49.6	− 33.973.2	− 33.973.2	− 57.1	− 69.36.0	− 36.069.3	− **42.8**	− 83.236.3	− 36.383.2	0.755	0.064
	% seated	86.9	105.8	80.9	87.0	107.8	83.5	**81.6**	99.9	68.6	0.380	–
MEP	cmH_2_O	86.09	52.66	104.86	89.50	64.31	102.65	**57.14**	27.81	110.34	0.435	0.057
	% seated	88.0	99.8	76.3	88.9	99.5	81.8	**83.8**	98.5	70.3	0.435	–

*FEV*_*1*_ forced expiratory volume in the 1 s, *FVC* forced vital capacity, *FEF*_*25-75*_ forced expiratory flow at 25 and 75% of the pulmonary volume, *FRC* functional residual capacity, *TLC* total lung capacity, *RV* residual volume, *ERV* expiratory residual volume, *IC* inspiratory capacity, *VC* vital capacity, *MIP* maximal inspiratory pressure, *MEP* maximal expiratory pressure, *%pred* percentage of predicted value, *25p* 25th percentile, *75p* 75th percentile.

Lung restriction was diagnosed in 3 (17%) subjects: 2 (20%) class 2 subjects and 1 (14%) class 3 subjects. The restriction was diagnosed using both reference equation systems.

When the mean normal population values were considered, both MIP and MEP were ~ 70% of the predicted value; while MIP and MEP values were far higher than the lower limit of normal (Table 2).

### Ventilatory and thoraco-abdominal pattern

**Figure 2:**
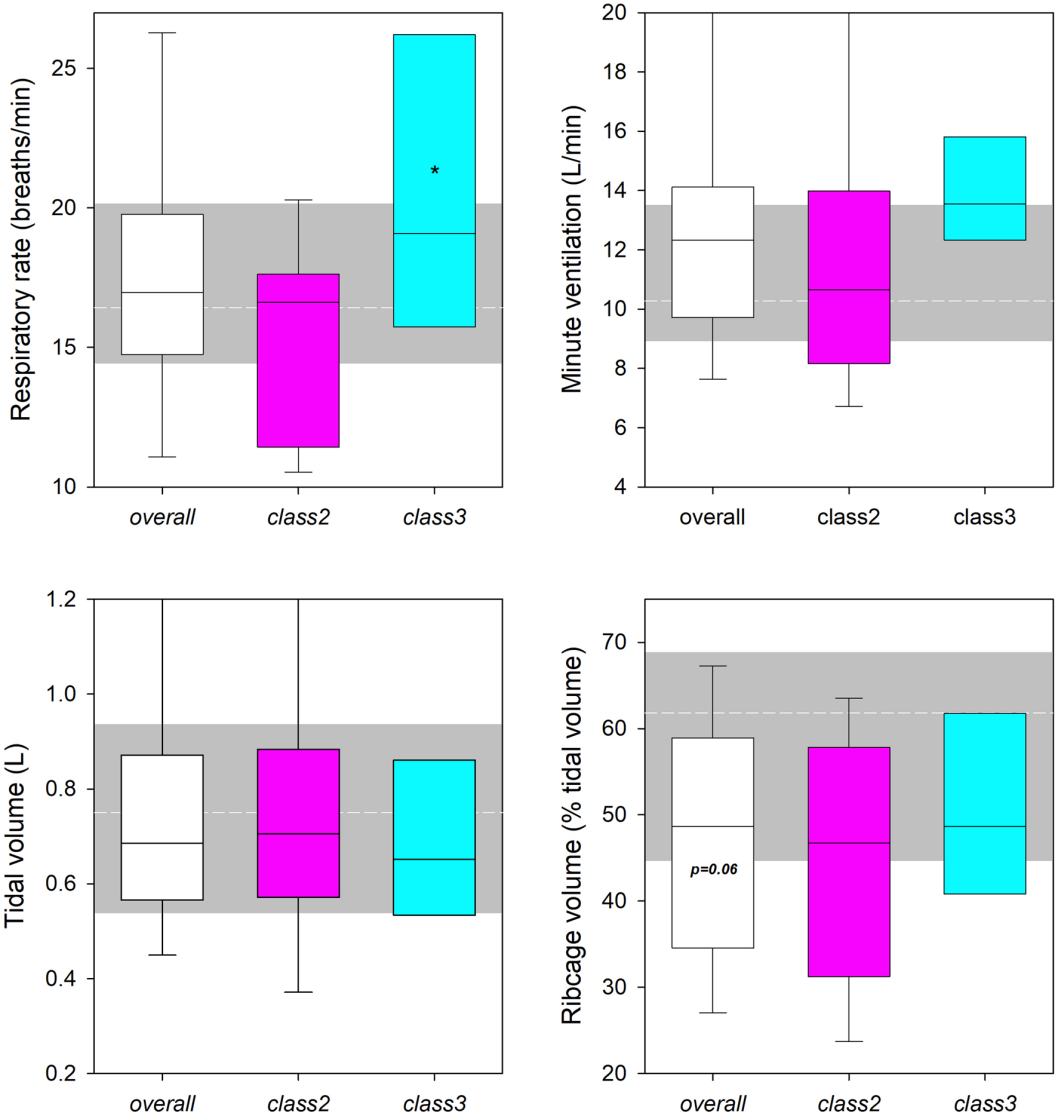
Seated position. Boxplot representing the median (line within the box), the interquartile range (the box), the 5th and the 95th percentile (bottom and top whiskers, respectively) of the respiratory rate (top left), minute ventilation (top right), tidal volume (bottom left) and its percentage ribcage contribution (bottom right) at rest during quiet breathing in overall (white box), class2 (pink box) and class3 (cyan dotted box) subjects with obesity. The short-dashed white line indicates the corresponding median value of the normal weighted subjects set as reference. The grey area indicates the corresponding interquartile range of the normal weighted subjects. *: *p* < 0.05 class 2 versus class 3.

**Figure 3:**
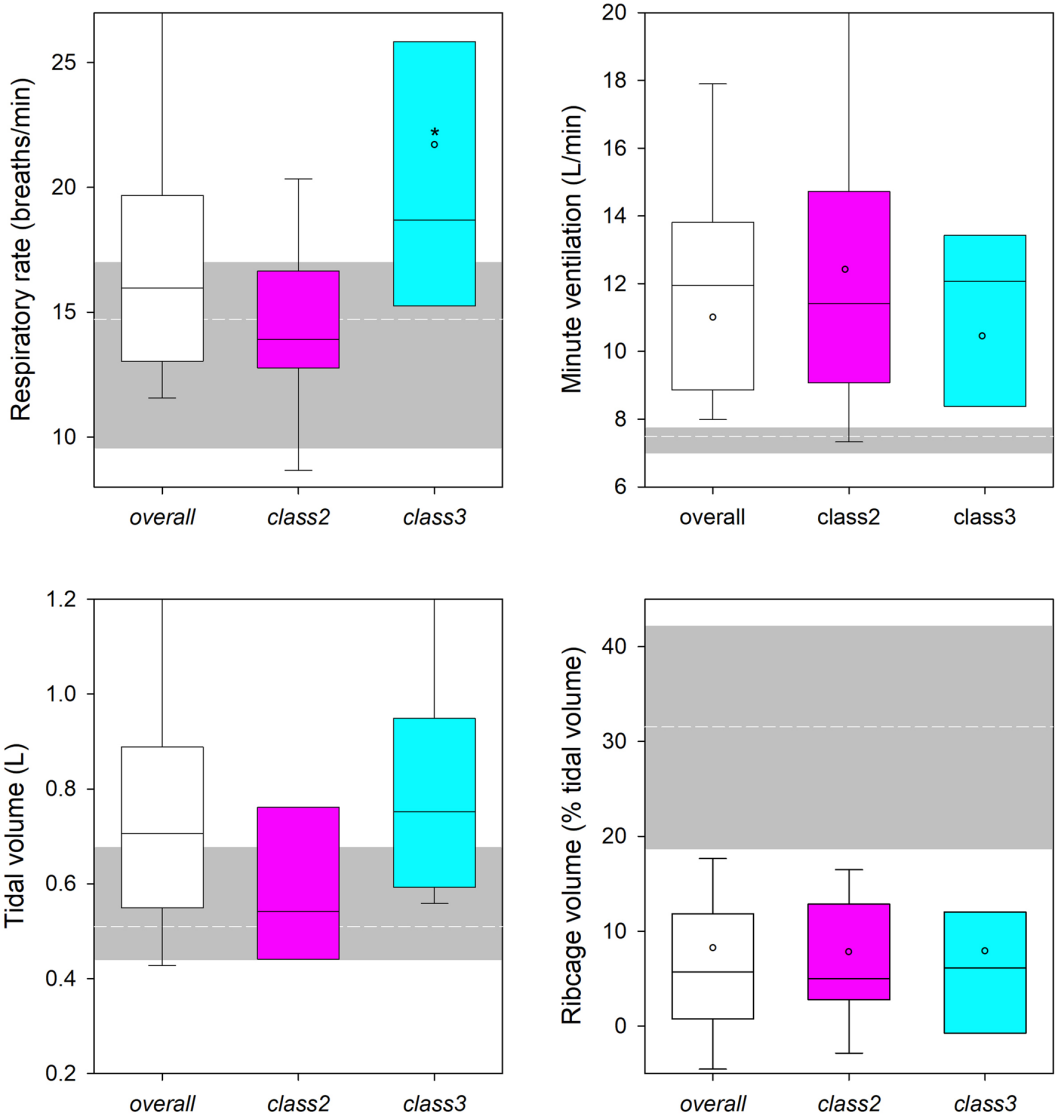
Supine position. Boxplot representing the median (line within the box), the interquartile range (the box), the 5th and the 95th percentile (bottom and top whiskers, respectively) of the respiratory rate (top left), minute ventilation (top right), tidal volume (bottom left) and its percentage ribcage contribution (bottom right) at rest during quiet breathing in overall (white box), class 2 (pink box) and class3 (cyan dotted box) subjects with obesity. The short-dashed white line indicates the corresponding median value of the normal weighted subjects set as reference. The grey area indicates the corresponding interquartile range of the normal weighted subjects. *: *p* < 0.05 class 2 versus class 3; °: *p* < 0.05 obese vs normal weighted subjects.

**Figure 4:**
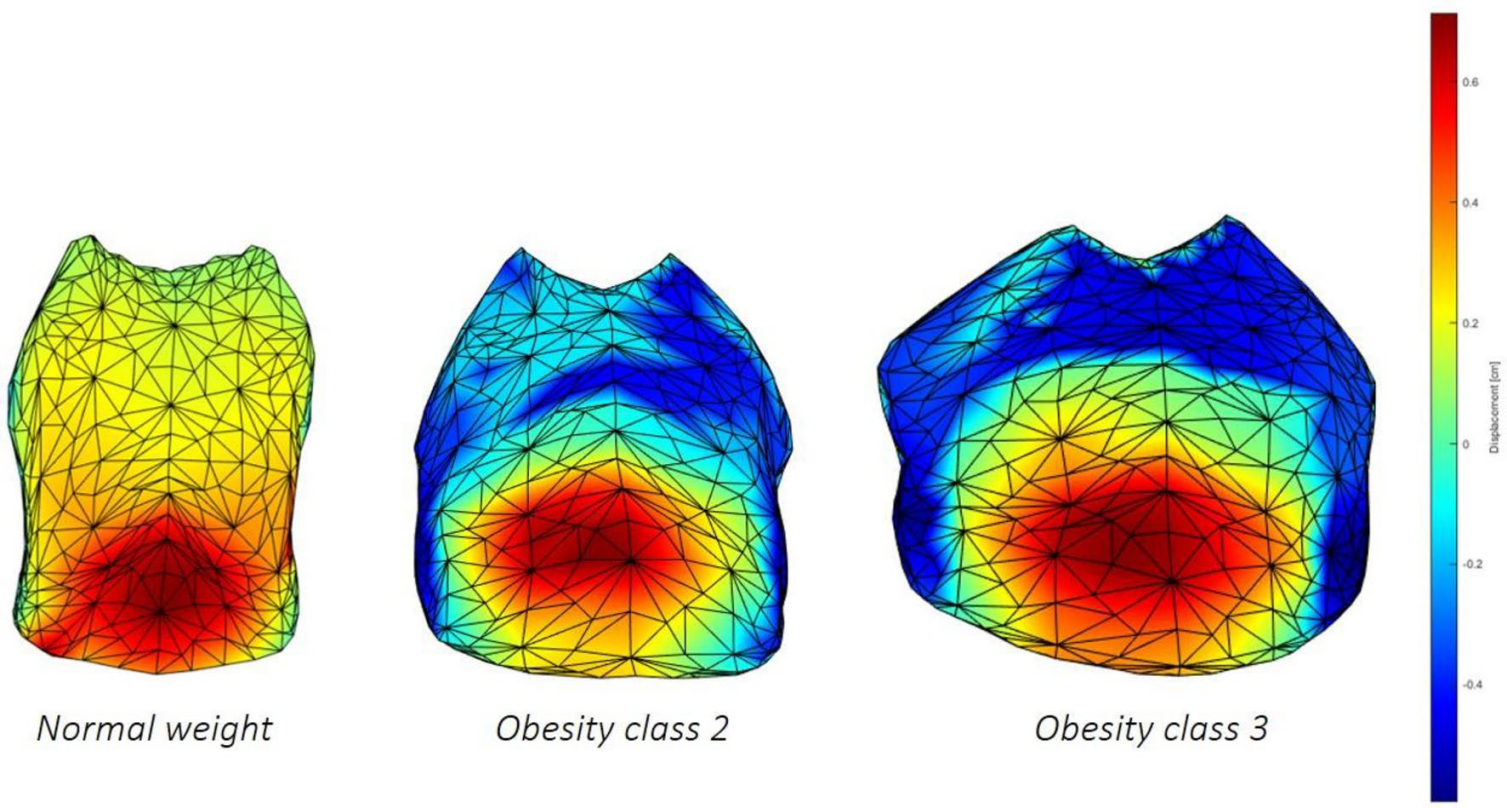
Colormaps of the magnitude of the trunk motion during tidal volume in the supine position in a represented normal weighted (left), class 2 (middle), and class 3subjects (right). The uniform color scale is also reported, with expansion ranging from blue (very low) to red (high)^38^.

In seated posture, the only parameter that was influenced by obesity class was breathing frequency, which was higher in class 3. The reduced ribcage percentage contribution to the tidal volume of overall obese subjects was close to the limit of significance (Fig. 2). Differences between subjects with obesity and those with normal weight occurred only lying supine, with the former breathing with higher minute ventilation and lower ribcage contribution to tidal volume, independently on the class of obesity. In addition, class 3 obese subjects breathed with a higher frequency rate also in the supine position (Fig. 3). Figure 4 provided with an immediate 3D graphic representation of the thoracic restriction in the supine position^38^.

### Correlations FRC-abdominal dimension

No significant correlation was found between the supine fall of FRC and the abdominal dimension.

## Discussion

We have shown that the accurate assessment of thoraco-abdominal contribution to tidal volume at rest in a supine position highlights a restrictive thoracic pattern that standard lung function tests do not relieve. We found a prevalence of lung restriction of 17%, while the supine ribcage expansion was systematically lower in almost all subjects with obesity. Our subjects with obesity showed relatively normal spirometry and lung volumes, as only residual and consequently expiratory reserve volumes were altered. Class 3 obesity affected the respiratory rate, which was higher during resting awake quiet breathing in both supine and seated position. Independently of the class, obesity altered the breathing pattern at rest in the supine position, in terms of higher ventilation and reduced thoracic contribution. The supine position also reduced the maximal pressures respiratory muscles can generate, although without indicating pathological weakness of respiratory muscles. As expected, the supine position induced an important fall of functional residual capacity but it was not correlated with the abdominal dimension. OEP volume overestimated the integrated flow by 8.5% at rest and by 20.2% during maximal maneuvers.

While traditionally being considered a problem only in high-income countries, nowadays overweight and obesity are dramatically rising in low- and middle-income countries, particularly in urban settings. For these reasons, it was of paramount importance to characterize the different obesity classes also according to the respiratory function and use new technique, like OEP, that might add additional or complementary information.

### Opto-electronic plethysmography

First, we have validated the method that we used to assess ventilatory function. Although partially validated^21^, OEP was recently proved to be a useful tool to evaluate the thoraco-abdominal kinematics of people with morbid obesity^17^. We have provided a complete validation considering both sexes, different tidal volumes, and two postures. Results are satisfactory and OEP proved to be viable in subjects within BMI = 55 (the maximal value in our subjects with obesity). More studies are therefore needed to demonstrate the feasibility of OEP in people with a BMI greater than this value^17^.

### Ventilatory and thoraco-abdominal pattern

The percentage contribution of the ribcage to the tidal volume was the parameter that mostly characterized the breathing pattern in subjects with obesity, being systematically lower than lean peers in the supine position. In the seated position, the value was still reduced, but close to the limit of significance (*p* = 0.059). However, a Brazilian study reported reduced ribcage expansion in seated position^21^, therefore reinforcing and supporting our results.

In addition, obese subjects had higher minute ventilation in the supine position.

### Spirometry, lung capacities, and maximal respiratory pressures

There is plenty of scientific literature on the effect of obesity on lung function. Obesity is reported to reduce FRC and ERV, with little effect on RV and TLC, resulting in slightly reduced FEV_1_ and FVC, with the FEV_1_/FVC ratio almost unaffected or increased (due to a decrease in the magnitude of FVC as compared to FEV_1_ because of the decreased compliance associated with the presence of fat deposits)^4,5,39–43^. Our results do not align with this, because only FEV_1_/FVC and RV were higher and ERV lower than expected. Since FVC, FEV_1_, TLC, and FRC were almost normal (not lower than 95% of the expected values) in our subjects with obesity, we failed to prove obesity to have a systematic restrictive effect on the lung in our small cohort of subjects with obesity.

This finding might seem in contrast with the pathophysiology of obesity. However, the percentage of restrictive defect that we found was in line with that reported by Collet and colleagues who showed ~ 15% of subjects with obesity having a restrictive defect^44^. In addition, other authors found normal spirometric and plethysmographic volumes in their subjects with obesity, with the term “normal” meaning within the expected predicted values^6,7,43,45–48^. Saliman et al. concluded that in morbid obesity restrictive ventilatory defects were less common than obstructive ventilatory patterns^49^.

Another interesting consideration is that the lung volume of our subjects with obesity was similar to those published by other authors^18,49,50^ when expressed in liters but not when expressed as a percentage of the predicted values. These lung volume and capacity results opened the discussion to the choice of lung function prediction equations, as this might introduce potential errors in the diagnosis. There is a plethora of published reference equations with most equations being based on small numbers of subjects. For example, in their interesting study on the effects of body mass index on lung volumes, Jones et al. used reference values based on only 627 subjects covering seven decades of age. Instead, we chose the Global Lung Function Initiative (GLI) sponsored and recommended by the European Respiratory Society, based on spirometric data of > 35 k subjects and modelled using modern statistical techniques^51^.

In addition, we found no difference between slow and forced vital capacity (*p* = 0.918) in our subjects with obesity. Such difference is supposed to increase with increasing body mass index^52^. Despite this, our subjects with obesity showed results compatible with the pathophysiology of obesity, except FRC. Reduced FRC in even mild obesity is very common, while we have found relatively normal FRC in our small group of subjects with obesity. Of note, the majority of the studies reported FRC only as a percentage^40,44,48^. The FRC values that we found were in line with those reported by two authors^8,49^ in terms of absolute values, but not of percentage values. Indeed, only Benedik and co-authors^7^ reported similar values of FRC percentage.

Taken together, in the absence of other respiratory pathology or condition or morbidity obesity seems not to have a systematical restrictive effect on the lung. This speculation is supported by a recent study that concluded that obesity affects pulmonary function in Japanese adult patients with asthma, but not those without asthma^53^. On the other hand, even in the absence of other respiratory pathology or condition or morbidity obesity seems to have a systematical restrictive effect on the ribcage. The accurate measurement of thoraco-abdominal volume, therefore, provided indications of obesity that were complementary to pulmonary function tests. In addition, OEP is able to localize the restrictive effect of obesity.

These considerations referred to BMI < 50 kg/m^2^ and they should be confirmed on a larger scale.

Maximal pressures were lower than the mean normal population values. However, in medical practice, the mean normal population values are of very little interest. To decide whether a patient has pathological weakness of respiratory muscles, the lower limit of normal is the parameter of interest. In this case, both MIP and MEP values were above the LLN. Both maximal pressures decreased in the supine position. In this posture, a thoracic restriction characterized the breathing pattern. Indeed, the ribcage contribution to tidal volume in supine position was lower than lean peers. In addition, in line with previously published results^18^, obesity determined higher minute ventilation in the supine position.

### Obesity class 2 versus class 3

Class 3 obesity, formerly known as morbid or severe obesity, not only contributes to potentially serious health problems but it is also associated with reduced economic and social opportunities and quality of life. For this reason, we have also searched for the impact of class 3 obesity on respiratory function.

Lung volumes were similar within the two groups of subjects with obesity. This finding did not surprise, as all subjects were characterized by abdominal obesity, also known as central obesity, with similar thoracic and abdominal dimensions. Indeed, the two classes differed only in the hip circumference. We can therefore speculate that the different BMI was mainly due to the extremities. In this way, the load on the thorax might be similar and therefore it did not affect lung function, volumes, and capacities. Other authors found BMI not influencing spirometry and lung plethysmography^40,48,49,54^, while others found (sometimes weak) overall correlation but not specifically for the highest classes^54,55^.

The original aspect of the present study was the detailed analysis of the ventilatory and thoraco-abdominal breathing pattern. Obesity classes seemed not to play an important role in the ventilatory outcome. The two classes of subjects with obesity differed only in terms of respiratory rate, in both seated and supine positions, being higher (although still within normal values^56^) in class 3. The increased respiratory rate was already found in subjects with obesity compared to lean peers^18,19,21,45^, but for the first time, we have shown this to be a particular feature of class 3 obesity.

### Postural change

The current findings highlighted that the body positions influenced lung volumes (in terms of reduced FRC and consequently increased IC and reduced ERV) and chest wall kinematics (in terms of increased ventilation and restrictive thoracic pattern) in asymptomatic abdominal obesity. The reduction in FRC and the increase in airflow resistance on adopting the supine posture are common features also in normal subjects^8^. FRC reduction is mostly due to the gravitational effects of the abdominal contents, resulting in the relaxed diaphragm taking a more expiratory (i.e.: cranial) position.

Finally, our failure to find any significant correlation between the absolute lung volume and the abdominal dimension was in line with Watson and collaborators^46^. They used MRI to measure internal and subcutaneous trunk fat as well as total abdominal and thoracic volumes. Their study also failed to support the hypothesis that restriction or impaired thoracic expansion was simply a consequence of a large abdominal volume or total trunk fat volume or its distribution^46^.

### Limits of the study

The lack of metabolic syndrome investigation as well as the categorization of adipose tissue located at the abdominal level into intra-abdominal fat and subcutaneous fat are important limits of the study. An impact of metabolic parameters (adiponectin) on the change of pulmonary function was proved in Chinese subjects with obesity ^47^. Visceral fat is known to be more metabolically active (than subcutaneous fat) and linked to the metabolic syndrome^57^. Metabolic syndrome, in turn, has been linked to asthma and impaired lung function in both adolescents and adults^39^. Another limit of the study was considering BMI up to 50 kg/m^2^. It would be of great interest to extend the same analysis to BMI > 50 kg/m^2^, in particular for the considerations on pulmonary function tests.

In conclusion, obesity seems to have a systematic restrictive pattern on the chest wall, more specifically on the ribcage, rather than on the lung and mostly in the supine position.

Otherwise healthy obesity might not be characterized by systematic restrictive lung patterns. Restrictive defect was present in 17% of subjects with obesity. Despite this, another sign of restriction could be poor thoracic expansion at rest in the supine position, resulting in increased ventilation. Class 3 obesity made respiratory rate further increased. Opto-electronic plethysmography and its thoraco-abdominal analysis of awake breathing add viable and interesting information in subjects with obesity that were complementary to pulmonary function tests. In addition, OEP is able to localize the restrictive effect of obesity.

## Data Availability

The datasets generated and/or analyzed during the current study are not publicly available due to respect local privacy laws but are available from the corresponding author on reasonable request.
